# Bleeding outcomes associated with rivaroxaban and dabigatran in patients treated for atrial fibrillation: a systematic review and meta-analysis

**DOI:** 10.1186/s12872-016-0449-2

**Published:** 2017-01-06

**Authors:** Pravesh Kumar Bundhun, Mohammad Zafooruddin Sani Soogund, Abhishek Rishikesh Teeluck, Manish Pursun, Akash Bhurtu, Wei-Qiang Huang

**Affiliations:** 1 Institute of Cardiovascular Diseases, the First Affiliated Hospital of Guangxi Medical University, Nanning, Guangxi 530027 People’s Republic of China; 2 Guangxi Medical University, Nanning, Guangxi 530027 People’s Republic of China; 3 Institute of Cardiovascular Diseases, the First Affiliated Hospital of Guangxi Medical University, Nanning, Guangxi 530021 People’s Republic of China

**Keywords:** Atrial fibrillation, Rivaroxaban, Dabigatran, Bleeding events, Intracranial bleeding, Gastrointestinal bleeding, Oral anticoagulants

## Abstract

**Background:**

Warfarin is commonly used as a secondary prevention of stroke in patients with atrial fibrillation (AF). However, limitations have been observed even with the use of this medication. Recently, several newer direct oral anticoagulants (DOACs) have been approved for use by the food and drug administrations. Unfortunately, these newer drugs have seldom been compared directly with each other. Therefore, this study aimed to compare the bleeding events associated with rivaroxaban and dabigatran in patients treated for non-valvular AF.

**Methods:**

EMBASE, Medline (National Library of Medicine) and the Cochrane Central Registry of Controlled Trials were searched for studies comparing rivaroxaban with dabigatran using the terms ‘rivaroxaban, dabigatran and atrial fibrillation’. Primary endpoints were: any bleeding outcomes, intracranial bleeding and gastro-intestinal (GI) bleeding. Secondary outcomes included stroke/systemic embolism (SE)/transient ischemic attack (TIA), venous thromboembolism and mortality. Odds ratios (OR) with 95% confidence intervals (CIs) were calculated. The pooled analyses were carried out with RevMan 5.3 software. All the authors had full access to the data and approved the manuscript as written.

**Results:**

A total number of 4895 patients were included. This analysis showed that rivaroxaban was not associated with a significantly higher bleeding event when compared to dabigatran (OR: 1.28, 95% CI: 0.95–1.72; *P* = 0.11). GI bleeding was similarly manifested between these two DOACs (OR: 0.98, 95% CI: 0.43–2.25; *P* = 0.97). Even if intracranial bleeding was higher with the use of rivaroxaban, (OR: 2.18, 95% CI: 0.51–9.25; *P* = 0.29), the result was not statistically significant. Moreover, stroke/SE/TIA and venous thromboembolism were also not significantly different (OR: 0.81, 95% CI: 0.53–1.23; *P* = 0.32) and (OR: 2.06, 95% CI: 0.73–5.82; *P* = 0.17) respectively. However, even if mortality favored dabigatran (OR: 1.42, 95% CI: 0.99–2.06; *P* = 0.06), this result only approached statistical significance.

**Conclusion:**

Head to head comparison showed that rivaroxaban was not associated with significantly higher bleeding events compared to dabigatran. Intracranial bleeding, GI bleeding, stroke/SE/TIA, venous thromboembolism and mortality were also not significantly different between these two DOACs. However, due to the limited number of patients analyzed, and which were mainly obtained from observational studies, this hypothesis might only be confirmed in future randomized trials. Furthermore, the CHADS_2_-VASC and HAS-BLED score which might play an important role in predicting bleeding risks should also not be ignored.

## Background

Warfarin is commonly used as a secondary prevention of stroke in patients treated for Atrial Fibrillation (AF) [1]. However, even if warfarin is used by majority of patients, it also has limitations. Recently, several new Direct Oral Anti-Coagulants (DOACs) have been approved for use by the Food and Drug Administrations [2]. Even if these DOACs have previously been compared to warfarin [3], they have seldom been compared with each other in systematic reviews. The indirect comparison of coronary risks associated with apixaban, rivaroxaban and dabigatran showed significant differences in safety outcomes with regards to acute coronary adverse events [4]. Moreover, a network meta-analysis showed apixaban and 110 mg dabigatran twice a day to be more effective in patients with non-valvular AF whereas dabigatran 150 mg bid might be preferable compared to rivaroxaban, for patients with a high risk of embolism [5]. However, direct head-to-head comparison of rivaroxaban and dabigatran has seldom been carried out. Therefore, this analysis aimed to compare the bleeding events associated with rivaroxaban and dabigatran in patients treated for AF.

## Methods

### Data sources and search strategy

English publications comparing rivaroxaban with dabigatran were searched from EMBASE, Medline (National Library of Medicine) and the Cochrane Central Registry of Controlled Trials using the terms ‘rivaroxaban, dabigatran and atrial fibrillation’. To enhance this search, the words ‘new oral anticoagulants’ and the abbreviation ‘AF’ were also used. Moreover, reference lists of potential articles were also searched for relevant studies.

### Inclusion and exclusion criteria

Studies were included if:

- They compared rivaroxaban with dabigatran.
- They consisted only of patients with AF.
- They reported bleeding events among their clinical endpoints.

Studies were excluded if:

- They did not compare rivaroxaban with dabigatran, but instead, compared them with other DOACs.
- They did not involve patients with AF.
- They did not report bleeding outcomes among their clinical endpoints.
- They were duplicates.

### Outcomes

The primary outcomes analyzed in this study were:

- Any bleeding event
- Intracranial bleeding
- Gastrointestinal (GI) bleeding

The secondary outcomes analyzed were:

- Stroke/systemic embolism (SE)/transient ischemic attack (TIA)
- Venous thromboembolism
- Mortality

Refer to Table 1 for more details.

**Table 1:** Reported outcomes

Studies	Reported outcomes	Follow up period	Types of patients
Gorst 2016 [8]	Stroke/SE/TIA, any bleeding, death, intracranial bleeding, GI bleeding, venous thromboembolism	1 year	-
Larock 2014 [9]	Stroke/TIA, venous thromboembolism, any bleeding, death	-	NVAF
Maura 2015 [10]	Bleeding events, ischemic stroke/SE	3 months	NVAF
Providencia 2014 [11]	Death, thromboembolism, stroke, TIA, SE, major bleeding, minor bleeding	30 days	-
Sherid 2014 [12]	Major bleeding other than GI bleeding, intracranial hemorrhage, stroke/TIA, venous thromboembolism, death, GI bleeding	40 days	-

*Abbreviations*: *SE* systemic embolism, *TIA* transient ischemic attack, *GI* gastrointestinal, *NVAF* non-valvular atrial fibrillation

### Data extraction and review

The studies were independently assessed by five authors (PKB, MZSS, ART, MP, AB). The total number of patients treated by rivaroxaban and dabigatran respectively, the year of patients’ enrollment, the type of study reported, the baseline features of the patients, the number of events reported, and the outcomes analyzed were systematically extracted. The bias risk was not assessed because this analysis only involved data obtained from observational studies [6]. During this data extraction process, any disagreement which occurred were discussed among the authors. However, if reaching a consensus was still difficult, a final decision was made by the sixth author (WQH).

### Statistical analysis

PRISMA (Preferred Reporting Items for Systematic Reviews and Meta-Analyses) [7] guideline was followed. Heterogeneity across the subgroups was assessed using the Cochrane Q-statistic test (*P* ≤ 0 · 05 was considered statistically significant) and the I^2^-statistic test. A fixed effects model (I^2^ < 50%) or a random effects model (I^2^ > 50%) was used depending on the value of I^2^.

Funnel plots were used to visually estimate publication bias.

We calculated Odds Ratios (OR) with 95% Confidence Intervals (CIs). The pooled analyses were carried out with RevMan 5.3 software. Ethical approval was not required for this type of research.

All the authors had full access to the data and approved the manuscript as written.

## Results

### Search result

**Fig. 1:**
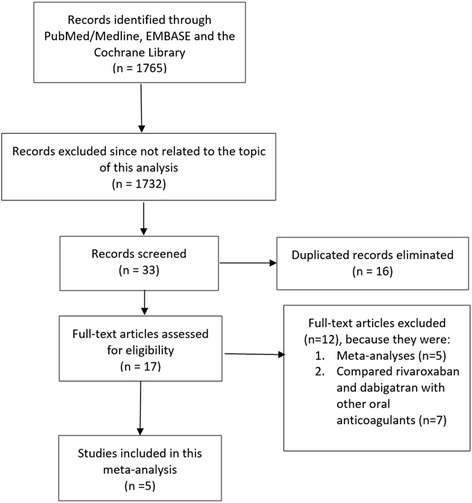
Flow diagram representing the study selection

A total number of 1765 articles were obtained during this search process. One thousand seven hundred and thirty-two articles were eliminated since they were not related to the topic of this search. Another 16 articles were eliminated since they were duplicates. Seventeen full-text articles were assessed for eligibility. However, further 12 articles were eliminated since: five articles were meta-analyses, whereas the remaining seven articles compared rivaroxaban or dabigatran with other anticoagulants (apixaban, warfarin). Finally, five articles were selected and included in this meta-analysis (Fig. 1).

### General features of the studies included

A total number of 4895 patients (1581 patients treated by rivaroxaban and 3314 patients treated by dabigatran) were included in this meta-analysis.

Table 2 summarizes the general features of the studies included.

**Table 2:** General features of the studies included

Studies	Type of study	Patients’ enrollment period	No of patients in Rivaroxaban group (n)	No of patients in Dabigatran group (n)
Gorst 2016 [8]	Observational	2012–2014	360	1190
Larock 2014 [9]	Observational	-	35	34
Maura 2015 [10]	Observational	2012	851	1687
Providencia 2014 [11]	Observational	2012–2013	188	176
Sherid 2014 [12]	Observational	2010–2013	147	227
Total no of patients (n)			1581	3314

Study Gorst 2016 included a very large number of patients. However, the other studies consisted of less patients. Therefore, in order for the result of this analysis not to be influenced by the result of study Gorst 2016, only patients with diabetes mellitus were selected from that particular study

### Baseline features of the studies included

The baseline features of the patients involved have been summarized in Table 3.

**Table 3:** Baseline features

Studies	Mean age	Males (%)	Ht (%)	Ds (%)	Cs (%)	DM (%)
	R/D	R/D	R/D	R/D	R/D	R/D
Gorst 2016 [8] Larock 2014 [9] Maura 2015 [10] Providencia 2014 [11] Sherid 2014 [12]	77.8/73.4 73.0/75.0 73.6/74.0 60.1/59.8 68.3/72.7	45.7/53.4 62.0/66.0 55.0/54.0 73.9/75.1 47.6/50.2	36.8/32.1 71.0/71.0 78.0/80.0 39.4/30.1 -	- - 47.0/47.0 - -	- - 3.0/4.0 - -	15.6/13.5 20.0/20.0 19.0/19.0 9.0/6.3 -

*Abbreviations*: *R* rivaroxaban, *D* dabigatran, *Ht* hypertension, *Ds* dyslipidemia, *Cs* current smoker, *DM* diabetes mellitus

The mean age of the patients ranged from 59.8 to 77.8 years. Dyslipidemia and current smoking were reported in only one study. Study Larock2014 had the majority of patients with diabetes mellitus. According to Table 3, there was no significant difference in baseline features among patients treated with rivaroxaban and dabigatran.

### Bleeding events associated with rivaroxaban and dabigatran

Results of this analysis have been summarized in Table 4.

**Table 4:** Results of this analysis

Outcomes analyzed	OR with 95% CI	*P* value	I^2^ (%)
Any bleeding Intra-cranial bleeding Gastrointestinal bleeding Stroke/SE/TIA Venous thromboembolism Mortality	1.28 [0.95–1.72] 2.18 [0.51–9.25] 0.98 [0.43–2.25] 0.81 [0.53–1.23] 2.06 [0.73–5.82] 1.42 [0.99–2.06]	0.11 0.29 0.97 0.32 0.17 0.06	0 0 0 0 0 38

*Abbreviations*: *SE* systemic embolism, *TIA* transient ischemic attack, *OR* odds ratios, *CI* confidence interval

**Fig. 2:**
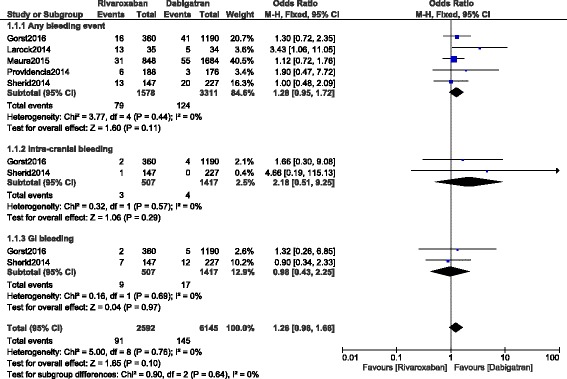
Bleeding events associated with rivaroxaban and dabigatran

This analysis showed that rivaroxaban was not associated with a significantly higher bleeding events compared to dabigatran (OR: 1.28, 95% CI: 0.95–1.72; *P* = 0.11, I^2^ = 0%). GI bleeding was similarly manifested between these two NOACs (OR: 0.98, 95% CI: 0.43–2.25; *P* = 0.97, I^2^ = 0%). However, even if intracranial bleeding was higher with rivaroxaban (OR: 2.18, 95% CI: 0.51–9.25; *P* = 0.29, I^2^ = 0%), the result was not statistically significant (Fig. 2).

### Other clinical outcomes reported between rivaroxaban and dabigatran

**Fig. 3:**
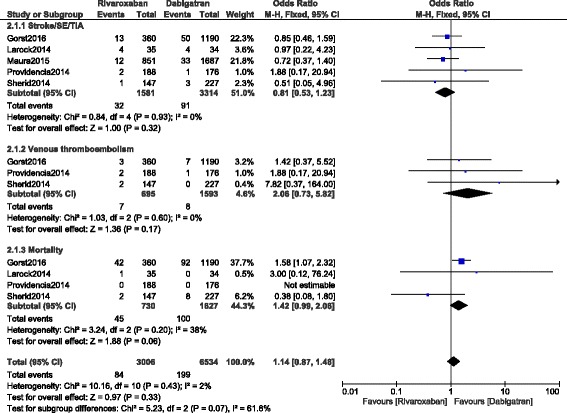
Adverse clinical outcomes associated with rivaroxaban and dabigatran

Apart from the bleeding events, other adverse outcomes were also analyzed. Stroke/SE/TIA and venous thromboembolism were not significantly different between these two groups (OR: 0.81, 95% CI: 0.53–1.23; *P* = 0.32, I^2^ = 0%) and (OR: 2.06, 95% CI: 0.73–5.82; *P* = 0.17, I^2^ = 0%) respectively. However, even if mortality favored dabigatran (OR: 1.42, 95% CI: 0.99–2.06; *P* = 0.06, I^2^ = 38%), the result only approached statistical significance (Fig. 3).

**Fig. 4:**
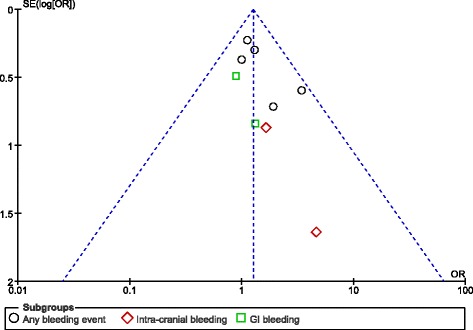
Funnel plots showing publication bias

**Fig. 5:**
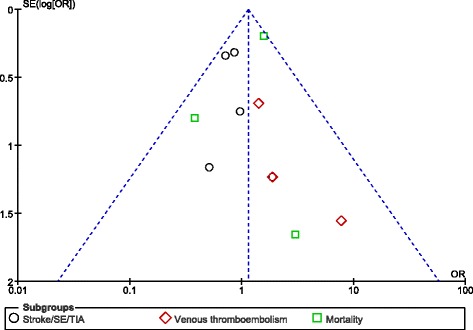
Funnel plots showing publication bias

For the above analyses, sensitivity analyses yielded consistent results. Based on a visual inspection of the funnel plots obtained, there has been only little evidence of publication bias for the included studies that assessed bleeding outcomes and the other clinical outcomes respectively (Figs. 4 and 5).

## Discussion

This analysis which aimed to compare (head to head) the bleeding outcomes associated with rivaroxaban versus dabigatran in patients treated for AF showed no significant difference in bleeding events, including intracranial and GI bleeding. Also, no significant difference was observed in stroke/SE/TIA and venous thromboembolism. However, mortality which was shown to favor dabigatran only approached significance.

Five observational studies were included in this current meta-analysis. The prospective cohort including patients with non-valvular AF recruited between February 2012 to August 2014 (with new users of 15 mg or 20 mg rivaroxaban respectively who were older and associated with more co-morbidities compared to new users with 110 or 150 mg dabigatran) showed rivaroxaban to be associated with a lower or similar proportion of stroke, but with an increased level of mortality and bleeding compared to dabigatran [8]. Another prospective study used in this current analysis and including 69 patients showed adverse events with the use of rivaroxaban and dabigatran respectively due to their inappropriate use and suggested collaboration with clinical pharmacists for better use of these drugs [9]. A French Nationwide Propensity-Matched Cohort Study which has been used in our current analysis showed similar bleeding and ischemic risks between rivaroxaban versus warfarin, and dabigatran and warfarin in the propensity-matched cohort [10]. In addition, another study including 556 consecutive patients showed both drug rivaroxaban and dabigatran to be effective and safe compared to warfarin [11]. Another head-to-head comparison on the other hand showed dabigatran and rivaroxaban to have a higher bleeding risk in the first 40 days, but dabigatran was not associated with an increased risk of GI bleeding [12].

Previously, the indirect comparison involving 27 randomized trials showed significant differences in the comparative safety of apixaban, rivaroxaban and dabigatran [4]. However, these DOACs were individually compared with warfarin whereby apixaban, rivaroxaban and dabigatran showed different results from this current analysis which compared rivaroxaban with dabigatran. Moreover, the network meta-analysis involving data from the RE-LY, ARISTOTLE and ROCKET AF trials, showed apixaban to be safer compared to rivaroxaban or dabigatran (150 mg) except that intracranial hemorrhage and all-cause mortality were similarly manifested [5].

A study based in Australia showed both oral anticoagulants to be associated with considerable amount of hemorrhage within the first 30 days [13]. Two hundred and forty hemorrhagic adverse events were observed with rivaroxaban whereas 504 events were observed among patients treated with dabigatran. But despite the fact that a higher rate of bleeding events was observed in both of the groups, rivaroxaban and dabigatran were barely compared.

Another study which included 444 patients with paroxysmal, persistent and longstanding-persistent AF showed comparable bleeding events between rivaroxaban and dabigatran [14]. Even in the study published by Fontaine et al, a comparable major bleeding events was observed between these two DOACs [15]. Similar to this current analysis, no significant difference in major complications was observed between these two DOACs. Also, a population based observational study showed a similar rate of GI bleeding associated with DOACs and warfarin [16] but the authors emphasized on the fact that cautions should be taken when prescribing DOACs to older patients.

In a recently published meta-analysis showing the impact of DOACs on GI bleeding in patients with AF, the authors concluded that rivaroxaban, edoxaban and higher dosages of dabigatran should be avoided in patients who are at higher risk of suffering from GI bleeding [17]. But the fact that their study only aimed to investigate GI bleeding associated with DOACs compared to warfarin should not be ignored and their conclusion was interpreted in comparison to warfarin whereas this current study compared rivaroxaban with dabigatran, instead of rivaroxaban with warfarin and dabigatran with warfarin respectively.

Coronary risk was also compared among the DOACs (dabigatran, apixaban and rivaroxaban), but even if a signal of increased coronary risk was observed with dabigatran, no direct comparison was made [18] implying that head to head comparison was a major limitation in that study.

Another meta-analysis published by Caldeira et al showed that DOACs might be as safe as warfarin in patients who were to be treated for non-valvular AF [19]. In addition, the study published by Yao et al, using patients from a large United States database, showed that in patients with non-valvular AF, dabigatran was associated with a similar rate of stroke, but with a lower rate of major bleeding when compared to warfarin [20]. Also, when warfarin and rivaroxaban were compared, the latter was associated with similar rate of stroke and major bleeding. The network meta-analysis comparing the effectiveness of interventions for stroke prevention in patients with AF also showed all oral anticoagulants to reduce stroke/systemic embolism and mortality [21].

Randomized trials have been conducted so far only to compare each of the DOACs with warfarin. However, no randomized trial has been conducted to show a head-to-head comparison between two different DOACs. There might be underlying market agreements aimed at avoiding conflicts of similar products, in view of the common goal, which is to subtract market space to Vitamin K antagonists.

DOACs might be cost-effective [22] but their benefits should be further studied for future care. The Canada-based study comparing rivaroxaban with dabigatran in patients treated for AF found dabigatran to be economically dominant for the prevention of stroke and SE [23]. The study by Miguel et al also showed dabigatran to improve clinical events with a lower cost, and it was preferred to rivaroxaban in Portuguese patients who suffered from AF [24].

However, these hypotheses will have to be further proved in newer randomized trials beginning with the PREFER-AF trial which should be the first randomized trial evaluating vascular protective effects of these DOACs [25]. The overall use of these DOACs will also depend on several factors such as patient satisfaction, cost, and risk profile of patients. Currently, it should not be ignored that the CHADS_2_-VASC score is used to assess patients for the risk of stroke whereas the HAS-BLED score is used to calculate the risk of bleeding in patients who are treated by oral anticoagulants [26, 27]. Renal function should also be taken into account before considering DOACs in patients with non-valvular AF [28].

### Novelty

The idea of this research is new in clinical medicine. Several DOACs have been approved for use in this new era. However, even if they have previously been compared with warfarin, they have seldom been directly compared with each other in systematic reviews and meta-analyses. By comparing rivaroxaban with dabigatran, this direct head to head analysis represents a new feature contributing to the literature of clinical medicine. Moreover, despite of including data obtained from observational studies, a very low level of heterogeneity was observed among all the subgroups analyzed which might be another new feature of this study.

### Limitations

This study also has limitations. First of all, due to the restricted number of patients, this analysis might not provide robust results. In addition, only data from observational studies were included. Since data obtained from observational studies are not expected to be as good as data obtained from randomized trials, another limitation might be considered. Heterogeneous data which were included might also affect the results. Moreover, when analyzing stroke/SE/TIA, two studies which did not include TIA and SE respectively, were also included in this subgroup and analyzed due to a lack of data. This might also affect the result of this analysis. The follow-up periods reported in each study involved were also ignored, further contributing to the limitations in this study.

## Conclusion

Head to head comparison showed that rivaroxaban was not associated with significantly higher bleeding events compared to dabigatran. Intracranial bleeding, GI bleeding, stroke/SE/TIA, venous thromboembolism and mortality were also not significantly different between these two DOACs. However, due to the limited number of patients analyzed, and which were mainly obtained from observational studies, this hypothesis might only be confirmed in future randomized trials. Furthermore, the CHADS_2_-VASC and HAS-BLED score which might play an important role in predicting bleeding risks should also not be ignored.

## Abbreviations

AF: Atrial fibrillation
CI: Confidence intervals
DOACs: New direct oral anticoagulants
GI bleeding: Gastrointestinal bleeding
OR: Odds ratios
SE: Systemic embolism
